# Au-TiO_2_/Ti Hybrid Coating as a Liquid and Gas Diffusion Layer with Improved Performance and Stability in Proton Exchange Membrane Water Electrolyzer

**DOI:** 10.3390/molecules27196644

**Published:** 2022-10-06

**Authors:** Gaoyang Liu, Shanlong Peng, Faguo Hou, Baizeng Fang, Xindong Wang

**Affiliations:** 1 Department of Energy Storage Science and Technology, University of Science and Technology Beijing, 30 College Road, Beijing 100083, China; 2 Department of Metallurgical and Ecological Engineering, University of Science and Technology Beijing, 30 College Road, Beijing 100083, China; 3 Department of Chemical and Biological Engineering, University of British Columbia, 2360 East Mall, Vancouver, BC V6T 1Z3, Canada

**Keywords:** proton exchange membrane water electrolyzer, liquid and gas diffusion layer, hybrid coating, corrosion resistance, interfacial contact resistance

## Abstract

The liquid and gas diffusion layer is a key component of proton exchange membrane water electrolyzer (PEMWE), and its interfacial contact resistance (ICR) and corrosion resistance have a great impact on the performance and durability of PEMWE. In this work, a novel hybrid coating with Au contacts discontinuously embedded in a titanium oxidized layer was constructed on a Ti felt via facile electrochemical metallizing and followed by a pre-oxidization process. The physicochemical characterizations, such as scanning electron microscopy, energy dispersive spectrometer, and X-ray diffraction results confirmed that the distribution and morphology of the Au contacts could be regulated with the electrical pulse time, and a hybrid coating (Au-TiO_2_/Ti) was eventually achieved after the long-term stability test under anode environment. At the compaction force of 140 N cm^−2^, the ICR was reduced from 19.7 mΩ cm^2^ of the P-Ti to 4.2 mΩ cm^2^ of the Au-TiO_2_/Ti. The corrosion current density at 1.8 V (RHE) is 0.689 μA cm^−2^. Both the ICR and corrosion resistance results showed that the prepared protective coating could provide comparable ICR and corrosion resistance to a dense Au coating.

## 1. Introduction

Hydrogen is green energy carrier, which has diverse applications. Green hydrogen can be produced via various technologies, such as electrocatalytic [1,2,3,4,5,6] or photocatalytic water splitting [7,8,9,10,11,12]. Proton exchange membrane water electrolyzer (PEMWE) is one of the important technologies for hydrogen generation. PEMWE has the advantages of high efficiency, environmental protection, excellent hydrogen quality, fast start-up, and strong power variability, and has become one of the best ways to develop the hydrogen energy economy [13,14,15].

Generally, there are two liquid/gas diffusion layers (LGDL) embedded on the both sides of the membrane electrode assembly (MEA) in PEMWE electrolytic cells. The main function of the LGDL is acting as a conductor, which can make electrons reach the cathode side from the anode through the external circuit, form a closed-loop circuit with the external power supply, and promote the rapid discharge of reaction products and water from the electrolytic cell. This requires that the LGDL material has good corrosion resistance, oxidation resistance, low interface contact resistance (ICR) and good hydrogen embrittlement resistance at the cathode at a high anode potential (>1.6 V vs. SHE) [16,17,18]. At present, the titanium substrates such as Ti felts and meshes are mainly used as LGDL on the anode side of PEMWE cells due to their excellent strength, low resistivity, high thermal conductivity and low permeability [19,20]. However, during the operation of the electrolytic cell, especially at the oxygen (anode) side, an oxide passivation layer will be gradually formed on the titanium surface, which greatly increases the ICR and reduces the overall performance [14,21].

At present, surface modification of the titanium LGDLs can effectively improve the corrosion resistance, electrical conductivity and ensure the service life [22,23]. There have been many studies on the surface modification of titanium LGDLs to form dense coatings with high conductivity and high corrosion resistance, such as tantalum [24], ceramic materials, e.g., titanium nitride [22,25] and titanium carbide [26] produced via various methods, e.g., physical vapor deposition (PVD) [27,28], chemical vapor deposition (CVD) [29], etc. Even though good corrosion resistance and high conductivity have been achieved for the above-mentioned coatings, the production processes and the complicated equipment required makes it complex, and the modified Ti LGDLs were prone to be oxidized during the long-term use [26]. Electroplating and electroless plating of precious metals Au and Pt can achieve extremely low ICR and high corrosion resistance [30,31]. However, due to the high cost of precious metals gold and platinum, it is not suitable for mass production. In order to reduce the use of the precious metals, thin and dense gold coatings have been developed in recent years. The prepared gold coating was about 3–10 nm, showing a ICR of 15–22 m Ω cm^2^, which reduced the ICR of the LGDLs and provided better corrosion resistance for the LGDLs [32]. However, the amount of gold coating was still high, the preparation cost was too high and the preparation process was cumbersome. Another method is the preparation of hybrid coating [33,34,35], such as conducting polymer/metals, carbon/metals, etc. Comparatively, the corrosion resistance of hybrid coatings has been verified to be good in a short time due to the physical barrier to the ion intrusion or the anodic protection from the passivated film. However, there were a lot of defects in the hybrid coating, and its actual corrosion resistance in the PEMWE environment needs to be investigated for a long time [36,37]. The coating peeling phenomenon could be more serious in the long-term test than other coatings. Therefore, reducing the use of the precious metals in the coating, increasing the adhesion between the precious metal coating and the titanium substrate, and developing a simple and scalable deposition technology are imperiously required. 

In this study, a novel hybrid coating with low ICR and high corrosion resistance is proposed. The precious metal Au contacts were discontinuously dispersed and embedded in the titanium oxidized layer via a facile electrochemical metallizing method followed by a pre-oxidization process [38,39], and the Au contacts could act as a conductor to replace the current dense Au coating on the whole surface. Both the distribution and morphology of the Au contacts were regulated, and various electrochemical characterizations were applied to investigate the effects of the distribution and morphology of the Au contacts on the ICR and corrosion resistance. Furthermore, the long-term stability of the as-prepared protective coating in a real PEMWE was also evaluated, which demonstrated promising ICR and corrosion resistance. 

## 2. Results and Discussions

**Figure 1:**
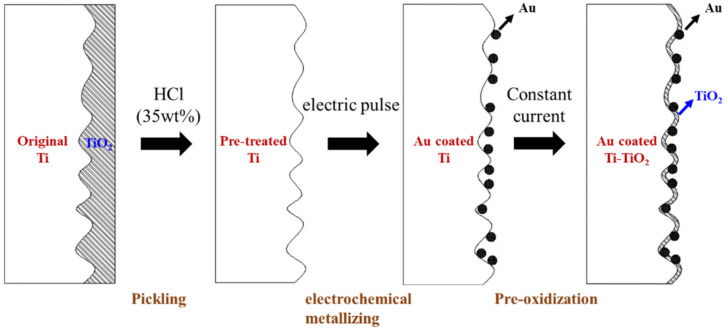
Schematic illustration of the formation of Au-TiO_2_/Ti hybrid coating.

The fabrication process for the novel hybrid protective coating composed of the Au contacts and TiO_2_ protective film constructed on a Ti felt is illustrated in Figure 1. First, prior to the deposition of the Au contacts on the surface of Ti felt, the Ti felt underwent thorough alkali cleaning and pickling in hydrochloric acid to remove the oxidized layer and form a rough and electronic conductive Ti surface (P-Ti). Then, the electrochemical metallizing process for the deposition of Au contacts on the P-Ti was carried out. Next, the Au contacts coated Ti felt (Au/Ti) was used in a PEMWE, and the titanium hydride layer around Au could be easily pre-oxidized into TiO_2_ on the anodic side during the long-term stability test (LTST). Thus, a hybrid coating consisting of the Au contacts embedded in the titanium oxidized layer (Au-TiO_2_/Ti) was eventually obtained. Compared with the current dense Au protective coating on the whole surface (Au/Ti (dense)), the Au-TiO_2_/Ti hybrid coating could be expected to have high corrosion resistance due to the Au and TiO_2_ hybrid coating. Meanwhile, the highly conductive and well dispersive Au contacts could provide enough electronic channels between the current collector and the LGDL, therefore decreasing the ICR. 

**Figure 2:**
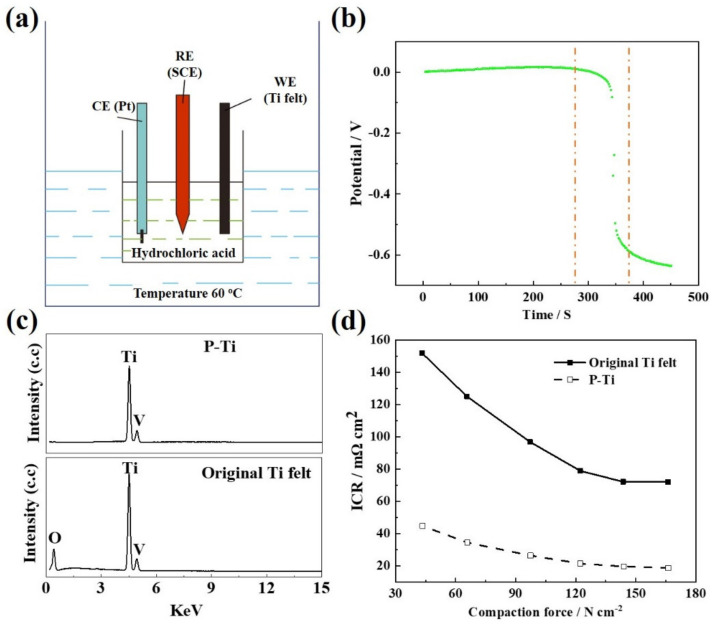
Schematic illustration of the three-electrode system for (**a**) in situ OCP recording, (**b**) the OCP change with time during the pickling process of Ti felt, (**c**) EDS spectrum, and (**d**) the relationship between ICR and compaction force of the original Ti felt and the P-Ti.

Generally, the presence of a passivation layer on the surface of Ti felt has a great negative impact on the ICR because the passivation layer itself is non-semiconductor [40,41]. In order to remove the oxidized layer, the Ti felts were pretreated via pickling in hydrochloric acid to form a rough and electronic conductive Ti surface. It should be noted that the OCP of the Ti oxides (e.g., TiO_2_ or other TiO_x_) is different from that of the pure Ti felt due to the different chemical states and reversible redox reactions which occur at the interfaces. Thus, the OCP of the Ti felts during the pickling in hydrochloric acid was in situ recorded in a traditional three-electrode system as shown in Figure 2a. Figure 2b shows the OCP change with time during the pickling process. Interestingly, when it was about 290 s (the left dash line in Figure 2b) of the pickling process, the OCP suddenly dropped from ca. −0.05 V to ca. −0.5 V. It indicated that the breakdown of the passivation layer (Ti oxides) on the surface of the Ti felts. After 390 s (the right dash line in Figure 2b), only a slight decay of the OCP was observed mainly ascribed to the further erosion of Ti surface. 

Figure 2c presents the EDS spectrum of the original Ti felt and the P-Ti. It can be further concluded that with the pickling pre-treatment of the original Ti felts, the oxidized layer was completely removed and a fresh Ti surface was formed. The surface composition change in the Ti felt could contribute to the lower ICR of the P-Ti than that of the original Ti felt as shown in Figure 2d. The complete removal of the passivation layer was of importance for the following electrochemical metallizing process for the deposition of the Au contacts on the felt. It can not only provide a clean surface for further growth of Au contacts, thereby improving the adhesion and stability of the coating, but also assures the fast electronic channels between the Ti felt and Au contacts.

**Figure 3:**
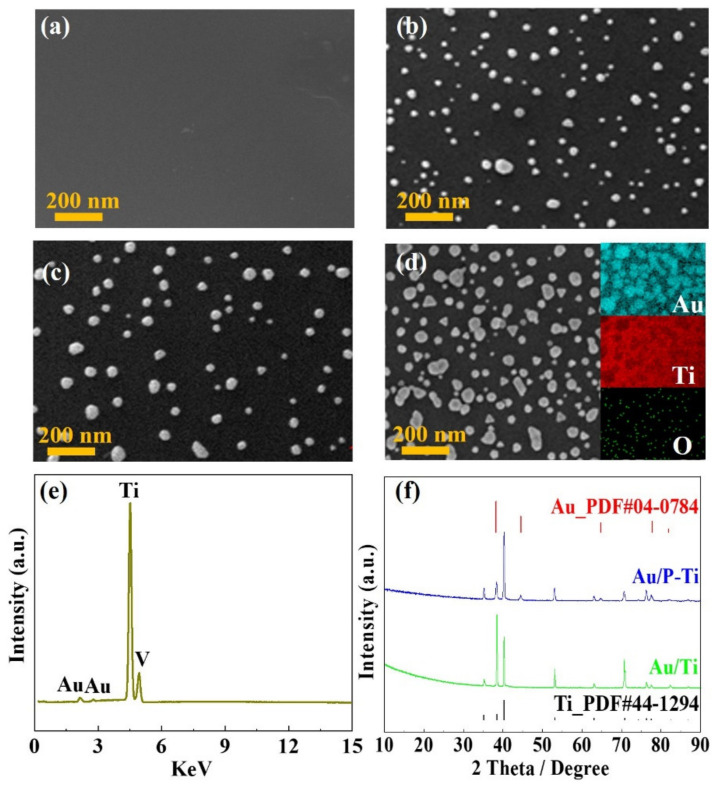
SEM images of (**a**) P-Ti, and Au/P-Ti prepared with different electrical pulse times: (**b**) 4 min, (**c**) 6 min and (**d**) 8 min, the insert is the corresponding EDS element mapping for Au, Ti and O, (**e**) EDS spectrum of the Au/Ti (6 min), (**f**) XRD patterns of the Au/P-Ti (6 min) and P-Ti.

The surface morphologies of the P-Ti and the Au/Ti felts prepared with the different electrical pulse times (i.e., 4 min, 6 min and 8 min) are shown in Figure 3a–d, respectively. It can be clearly found that the surface of the titanium felts did not form a dense gold coating, but the Au particles were uniformly distributed in a granular dotted manner (i.e., Au contacts). As the electrical pulse time increased, both the number and the size of Au contacts on the surface of the titanium felts increased. When the electrical pulse time was 4 min, there were fewer Au contacts and the size of the Au contacts were around 20–40 nm. Meanwhile, there were some small Au nodules on the surface of the P-Ti. When the electrical pulse time was 6 min, the size of the Au contacts increased to around 50 nm, but no uneven and smaller clumps appeared. When the electrical pulse time was 8 min, the Au contacts showed obvious coalescence phenomenon, and the unevenness of the surface could be observed due to a large number of the deposited Au contacts. The insert in Figure 3d presents the corresponding EDS element mapping for Au, Ti and O, and it revealed that the Au contacts were evenly distributed and coated on the surface of the P-Ti. It should be noted that almost no oxygen existed there, indicating that there was no oxidization during the Au coating process, and the atomic ratio of Au was around 2.6% for the Au/Ti (6 min) based on the EDS spectrum (Figure 3e). The composition of the Au/Ti (6 min) was determined to be Au and Ti by the XRD pattern as shown in Figure 3f. It should be also noted that the size, number, and distribution of the Au contacts could significantly affect the electronic channels, and result in difference in the ICR. In addition, it can be deduced that the coverage of the Au contacts could also affect the corrosion resistance of the Ti felt, which was further studied later. 

**Figure 4:**
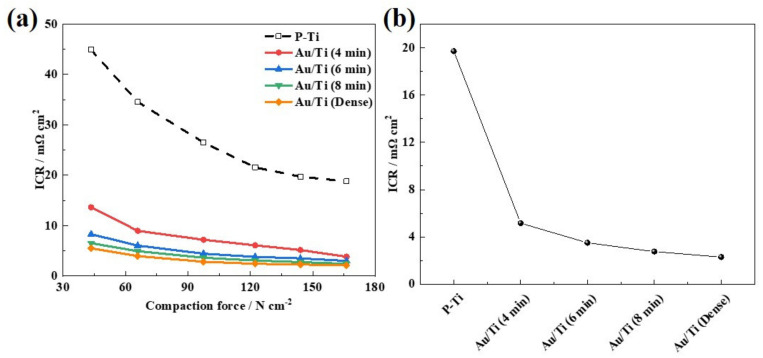
(**a**) Relationship between ICR and the compaction force, (**b**) ICR at 140 N cm^−2^ of the P-Ti and the Au/Ti prepared with different electrical pulse times.

The ICR between the LGDL and BP directly affects the power density and performance of the PEMWE devices [31,32]. Therefore, the ICR test was conducted and the results are shown in Figure 4a. The ICR values of the different samples at 140 N cm^−2^ (a common compaction force in PEMWE) are plotted in Figure 4b and also summarized in Table 1. It can be seen that the ICR of the samples gradually decreased with the increasing compaction force due to the gradual increase in the contact area between the sample and gold-plated BP. When the compaction force was above 140 N cm^−2^, the ICR of the samples stabilized with the increase in pressure and no longer changed significantly due to the full contact. As mentioned above, although the low-conductive oxide film on the surface of the Ti felt was removed by the acidic pickling, the ICR of the P-Ti at a compaction force of 140 N cm^−2^ was still large, about 19.7 mΩ cm^2^. Interestingly, the ICR of the Au/Ti samples with an Au contacts coating was significantly reduced (lower than 10 mΩ cm^2^). This is attributed to the Au contacts’ ability to provide fast electronic channels between the Ti felt and the BP. It should be noted that with the increasing size and number of the Au contacts, the ICR under the compaction force of 140 N cm^−2^ would be decreased with the increased electrical pulse time. Typically, the Au/Ti samples prepared with an electrical pulse time of 6 min (3.5 mΩ cm^2^) and 8 min (2.8 mΩ cm^2^) could reach a comparable ICR to the dense Au/Ti (2.3 mΩ cm^2^). Overall, the ICR between LGDL and BP was significantly reduced through the Au contacts coating, which is extremely meaningful for reducing ohmic losses in PEMWE.

**Figure 5:**
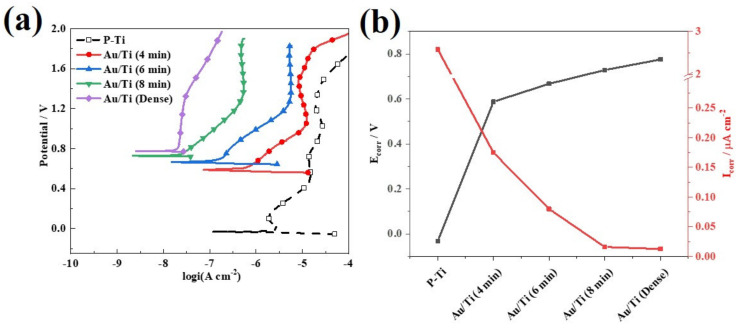
Potentiodynamic test results of the P-Ti and Au/Ti prepared with different electrical pulse times: (**a**) polarization curves; (**b**) *E*_corr_ and *I*_corr_ curves.

In the acidic and high-potential environment of PEMWE, the corrosion resistance is also an important indicator [16,41,42]. The corrosion resistance of the different samples was further evaluated in simulated PEMWE cells. The potentiodynamic polarization curves of the P-Ti and the Au/Ti prepared with different electrical pulse times (4 min, 6 min and 8 min) are shown in Figure 5a. Tafel fitting was performed in the strong polarization region in the polarization curve to further study the corrosion resistance change before and after the Au contacts coating, and the obtained corrosion potential *E*_corr_ and corrosion current density *I*_corr_ are shown in Figure 5b and also summarized in Table 1. It can be seen that with the increase in the electrical pulse time, the *I*_corr_ of all Au/Ti samples was much lower than the 2020 DOE target (below 1 μA cm^−2^) [43], and Au/Ti (8 min) exhibited the lowest *I*_corr_ (0.016 μA cm^−2^), which indicates that the corrosion resistance under the preparation condition was also optimal. From the surface morphology, it can be seen that with the increase in the electrical pulse time, both the surface coverage and the size of Au contacts increased, which can significantly improve the corrosion resistance of the Ti felt.

Based on the above data and analysis, it can be concluded that there was no need to form a dense Au coating, and both the Au/Ti (6 min) and Au/Ti (8 min) could provide comparable ICR and the corrosion resistance to the dense Au/Ti. However, the uncoated Ti area can be oxidized in the acid, oxygen-contained and high potential environment on the anode of PEMWE. Therefore, in order to investigate the effect of the oxidation of the uncoated Ti area on the surface composition as well as the ICR and the corrosion resistance of Au/Ti, the prepared Au/Ti (6 min) was further pre-oxidized in a simulated PEMWE cell.

**Figure 6:**
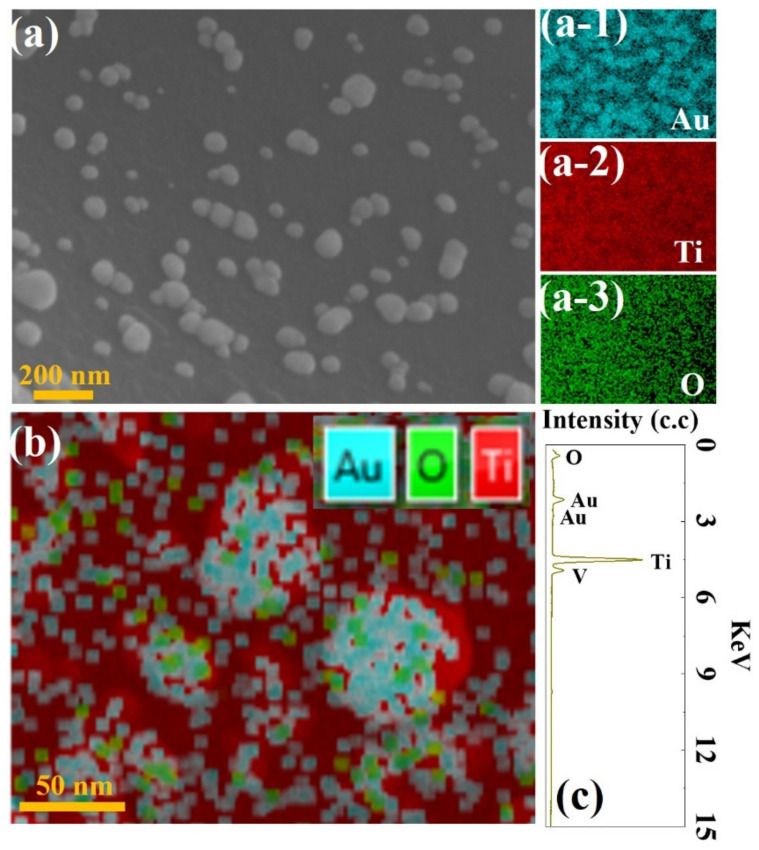
(**a**) SEM image and (a-1, a-2, a-3) the corresponding EDS elemental mappings for Au, Ti and O, (**b**) overlapped EDS elemental mapping for Au, Ti and O, (**c**) EDS spectrum of the Au/Ti (6 min) after the LTST.

Figure 6 presents the SEM images and the corresponding EDS mappings of the Au/Ti (6 min) after the long-term stability test (LTST). It can be clearly seen that the Au contacts maintained the granular dotted structure and were uniformly distributed, while the size of the Au contacts slightly increased compared with the Au/Ti (6 min) before the LTST. Figure 6a-1, a-2, and a-3 present the corresponding EDS element mapping of the Au/Ti (6 min). The elements of Au, Ti and O were observed after the LTST, where the O overlaid mainly with the Ti as shown in Figure 6b. It confirmed that only the uncoated area without the Au contacts appeared to be oxidized, where the oxygen atomic ration significantly increased from near 0 to 10.2% from the EDS spectrum of the Au/Ti (6 min) after the LTST, as shown in Figure 6c. Overall, it can be concluded that the Au-TiO_2_/Ti hybrid coating consisting of the Au contacts and TiO_2_ were eventually established on the Ti felt. The Au contacts not only provided the electronic channels, but also contributed the superior corrosion resistance together with the oxidized TiO_2_ film.

**Figure 7:**
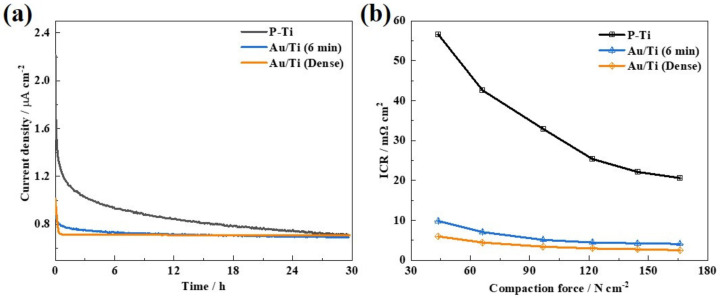
(**a**) Chronoamperrometry curves of the Au/Ti (6 min), (**b**) ICR at 140 N cm^−2^ after the LTST of the P-Ti, Au/Ti (6 min) and Au/Ti (dense).

Figure 7a shows the LTST results with chronoamperometry tests of the Au/Ti (6 min) for 30 h under the simulated PEMWE anode environment (+1.8 V, 0.5 M H_2_SO_4_ + 2 ppm F^-^ solution, O_2_ bubbling, ambient temperature). It can be seen that both the P-Ti and Au/Ti (6 min) showed a sharp drop at the beginning followed by a gradually decreasing process in the current density, which could be related to the formation of new protective oxide film on the Ti surface under the acidic, oxygen-contained and high-potential environment [16,32,44]. However, the current density of the P-Ti was much higher than that of the Au/Ti (6 min), indicating that the coated Au contacts could suppress the oxidizing process. Whereas, the current density of the dense Au coating maintained a stable state throughout the LTST, illustrating the excellent long-term stability of the Au/Ti (dense). Compared with the Au/Ti (dense), both the P-Ti and Au/Ti (6 min) can achieve comparably low corrosion current density after the LTST, indicating that the formation of the protective oxide film can enhance the corrosion resistance, resulting in the less dissolution of metal during the LTST. Figure 7b presents the ICR results of the samples after the LTST (also listed in Table 1). It is clear that the ICR of the P-Ti significantly increased after the LTST mainly due to the formation of the Ti oxide layer [23]. Even though both the P-Ti and Au/Ti (6 min) could provide effective corrosion resistance, the ICR of the P-Ti after the LTST was still extremely high and cannot meet the 2020 DOE target [43]. However, the ICR of the Au/Ti (6 min) after the LTST only slightly increased, and was still below 10 mΩ cm^2^ mainly due to the fast electronic channels from the Au contacts. Therefore, it can be concluded that the construction of Au contacts on Ti felt via the electrochemical metallizing process could be an effective strategy for producing LGDL with superior corrosion resistance and high stability.

## 3. Experimental

### 3.1. Materials

All chemical reagents were purchased from Sinopharm Chemical Reagent Beijing Co., Ltd., Beijing, China and were used as received without further purification in this work. 

### 3.2. Pickling Pre-Treatment and Electrochemical Metallizing

Commercial Ti felts (Ti, 99.7%, 0.36 mm thick) were used as the original material of the LGDL in the experiment, and were cut into 1 cm × 2 cm sheets for the electrochemical metallizing process. For the pretreatment [23], the Ti felts were firstly cleaned with acetone and ethanol. Secondly, they were immersed in deionized water and sonicated for 10 min to remove any mechanical impurities and completely submerge their porous structure. Thirdly, the Ti felts were dropped into 35 wt% hydrochloric acid solution (refluxed and preheated to 54 °C) for 15 min. Finally, the etched Ti felts were thoroughly washed with deionized water to remove the unreacted hydrogen chloride. The rinsed Ti felts were dried and then used for further electrochemical metallizing process. The pickling-pretreated Ti felts are marked as P-Ti.

The electrochemical metallizing process for the deposition of Au on the Ti felts was carried out with a home-made setup, where a graphite rod with a tip area of 0.5 cm^2^ acted as the brush anode and the pickling-pretreated P-Ti as the cathode. The electrolyte solution was commercial HAuCl_4_ solution with a pH value of 2.3. 

VMP2 electrochemical workstation was used to apply electric pulse. During the electrochemical metallizing process, the HAuCl_4_ solution was piped in between the gap of the brush anode and the P-Ti cathode, and then 100 mA peak current of electric pulse was applied, the on-off ratio of the electric pulse was 1:10, the on-off time t_on_ and t_off_ were 10 ms and 100 ms, respectively. The total electric pulse time was controlled to be 2 min, 4 min, 6 min and 8 min. After the electric pulse ended, the sample was taken out and washed with deionized water. Finally, the Ti felts with Au contacts on the surfaces were obtained and marked as Au/Ti (2 min, 4 min, 6 min), respectively.

### 3.3. Microstructure and Composition Analysis

Scanning electron microscopy (SEM) and energy dispersive spectrometer (EDS) were employed to analyze microstructure and cross-sectional features of the specimens with a Zeiss SUPRATM55 at an acceleration voltage of 15 kV. Powder X-ray diffraction (XRD) spectra were recorded on a RIGAKU D/max 2200 PC diffractometer with a graphite monochromator and Cu KCT radiation source (λ = 0.15418 nm). The content of metal elements was analyzed by an inductively coupled plasma atomic emission spectrometer (ICP-AES).

### 3.4. Electrochemical Measurements

The electrochemical tests were carried out using VMP2 (Bio-logic, Pairs, France) multi-channel electrochemical workstation under a three-electrode system. Graphite electrode was selected as the counter electrode (CE), saturated calomel electrode (SCE) as the reference electrode (RE), and the sample connected with platinum electrode clamp as the working electrode (WE). The tests were performed in a solution of 0.5 M H_2_SO_4_ + 2 ppm F^-^ solution in a simulated electrolytic cell environment at room temperature. Prior to each electrochemical test, the WE was maintained at the open circuit potential (OCP) for 30 min to make the potential reach a stable state. Unless otherwise specified, the potential involved in this study refers to the potential relative to SCE. The starting scanning potential of potentiodynamic polarization test was set to −0.1 V (vs. OCP). For higher potential conditions of PEMWE anode, the ending scanning potential was set to 1.6–2.0 V, and the scanning rate was set as 0.333 mV min^−1^. An electrochemical impedance spectroscopy (EIS) test was conducted in a frequency range of 100 kHz to 10 mHz with a perturbation amplitude of 10 mV under OCP conditions, and the EIS test results were fitted by ZSimpwin software. Moreover, O_2_ was blown into test solution to simulate PEMWE anode environment, and then a 12 h potentiostatic polarization test (long-term stability test) was implemented at ambient temperature and +1.6 V (vs. SCE) potential conditions. It is worth noting that all specimens need to be rinsed with deionized water and a new corrosive solution should be applied as the test environment before each test.

### 3.5. ICR Measurements

The LGDL is usually connected with the metal bipolar plate (BP) and the catalyst layer. The ICR should be minimized to improve the efficiency of PEMWE cell. The ICR between BP and diffusion layer was measured as reported by Wang et al. [23]. The sample used for the ICR measurement was located between two gold-plated copper plates, and a controllable uniform compaction force was applied on the top metal plate which was controlled by the CMT4103 electronic universal testing machine program. In addition, an AT515 precision resistance meter was used to measure the resistance of the entire circuit under different compaction forces. *R* includes the bulk resistance (2*R*_Cu_) of the two copper plates, the bulk resistance (*R*_DL_) of a titanium felt diffusion layer, and the contact resistance (2*R*_Cu/DL_) between the two copper plates and the diffusion layer; *R* can be expressed as:

(1) $$R=2R_{\mathrm{cu}}+R_{\mathrm{DL}}+2R_{\mathrm{Cu}/\mathrm{DL}}$$

Since the bulk resistances of gold-plated copper plate and the Ti felt samples were relatively small, *R*_DL_ and *R*_Cu_ could be ignored, and the calculated ICR was slightly larger than the actual value.

## 4. Conclusions

In this work, the surface of Ti felt was modified by a series of steps including acidic pickling, electrochemical metallizing and a pre-oxidization process to improve the corrosion resistance and conductivity of titanium felt as a LGDL. The structural characterizations with SEM, EDS, and XRD confirmed that a novel hybrid consisting of discontinuously distributed Au contacts and a titanium oxidized layer was eventually formed on the Ti felt (Au-TiO_2_/Ti). The distribution and morphology of the Au contacts can be regulated with the electrical pulse time, which can affect the corrosion resistance and ICR. It can be concluded that there is no need to form a dense Au coating. The as-prepared Au-TiO_2_/Ti samples could provide comparable ICR and the corrosion resistance to the dense Au coating. Typically, the Au-TiO_2_/Ti (6 min) revealed the ICR of only 4.2 mΩ cm^2^ at 140 N cm^−^^2^ and the *I*_corr_ of 0.080 μA cm^−2^. The chronoamperometry test results also confirmed the long-term stability of the as-prepared coating under the simulated anode environment. Meanwhile, the sample exhibited a comparable low current density to the Au/Ti (dense), which was stable at around 0.689 μA cm^−2^. Overall, the surface modification via the novel hybrid coating not only improved the corrosion resistance, but also decreased the contact resistance, and exhibited long-time stability as well. This work is proven to enrich the technologies of Ti-based BPs applied for PEMWEs.

## Figures and Tables

**Table 1:** Corrosion resistance and ICR of the different samples.

Specimens	Corrosion Resistance			ICR at 140 N cm^−2^	
	*E*_corr_ (mV)	*I*_corr_ (μA cm^−^^2^)	*I*_+1.8 V_ 30 h (μA cm^−2^)	ICR_Before_ (mΩ cm^2^)	ICR_After_ (mΩ cm^2^)
P-Ti	−0.032	2.580	0.718	19.7	22.1
Au/Ti (4 min)	0.587	0.175	-	5.2	-
Au/Ti (6 min)	0.667	0.080	0.689	3.5	4.2
Au/Ti (8 min)	0.728	0.016	-	2.8	-
Au/Ti (dense)	0.775	0.013	0.709	2.3	2.7

## Data Availability

Data will be available upon request from the corresponding authors.
